# A Three-Arm, Tiered Comparability Strategy Bridging Post-Approval Process Changes for an Omalizumab Biosimilar (CMAB007)

**DOI:** 10.3390/ph19050724

**Published:** 2026-05-02

**Authors:** Chenguang Wang, Chaoxin Zhou, Sheng Hou, Wenqiang Fan, Weizhu Qian, Yule Ren, Xiyuan Chen, Chenhong Pan, Qingcheng Guo, Huaizu Guo, Yajun Guo

**Affiliations:** 1 State Key Laboratory of Macromolecular Drugs and Large-Scale Preparation, School of Pharmaceutical Sciences, Wenzhou Medical University, Wenzhou 325035, China; 2 State Key Laboratory of Macromolecular Drugs and Large-Scale Preparation, NMPA Key Laboratory for Quality Control of Therapeutic Monoclonal Antibodies, Shanghai Zhangjiang Biotechnology Co., Ltd., Shanghai 201203, China; 3 State Key Laboratory of Macromolecular Drugs and Large-Scale Preparation, School of Pharmaceutical Sciences and Food Engineering, Liaocheng University, Liaocheng 252000, China; 4 Taizhou Mabtech Pharmaceuticals Co., Ltd., Taizhou 225300, China

**Keywords:** three-arm comparability strategy, post-approval changes, CMAB007, omalizumab biosimilar

## Abstract

**Background:** Post-approval manufacturing changes for biologics require rigorous comparability assessments to ensure uninterrupted quality and clinical performance. CMAB007 (Aomaishu^®^), a China-approved (2023) omalizumab biosimilar, underwent process enhancements—including media optimization and anion-exchange chromatography substitution—yielding a 5-fold increase in production without altering the host cell line. **Methods:** A novel three-arm tiered strategy was adopted to compare post-change CMAB007, pre-change CMAB007, and reference (Xolair^®^) products. Critical quality attributes (CQAs) were classified into tiers based on risk impact, with tier-specific acceptance criteria. Comprehensive analytics assessed structure, post-translational modifications, purity/impurities, activity, and Fc-mediated functions. Forced degradation (lyophilized/reconstituted states) and accelerated stability studies were evaluated. Based on the high degree of CMC similarity and to prevent “biological drift”, the pharmacokinetic (PK) and safety comparability of the post-change CMAB007 versus the reference product (Xolair^®^) was confirmed in a randomized, double-blind, two-arm study in healthy males (*N* = 114; single 150 mg subcutaneous administration). The pre-change product was not included in this clinical PK study. **Results:** Post-change CMAB007 exhibited analytical similarity within tiered acceptance criteria for all CQAs. Stability studies confirmed enhanced robustness under stress conditions. PK equivalence was demonstrated for AUC_0–inf_ (GMR: 99.82%; 90% CI: 91.46~108.94%), AUC_0–t_ (99.54%; 91.40~108.41%), and C_max_ (101.88%; 95.21~109.01%). Immunogenicity (ADA incidence: 10.5% vs. 12.5%, *p* = 0.742) and safety profiles were comparable. **Conclusions:** This study pioneers a tiered three-arm comparability strategy for post-approval changes, integrating advanced analytics, risk-based quality assessment, and clinical validation. The approach mitigates “biological drift” risks, ensuring biosimilar quality, efficacy, and safety while enabling sustainable production scalability.

## 1. Introduction

Omalizumab (Xolair^®^) is a humanized monoclonal antibody that specifically binds to immunoglobulin E (IgE), inhibiting its interaction with the high-affinity IgE receptor (FcεRI) on mast cells and basophils [1]. This mechanism underlies its therapeutic efficacy in allergic asthma and spontaneous urticaria, achieved by preventing the release of inflammatory mediators and downregulating FcεRI expression [2,3,4,5,6,7,8]. The inherent complexity of biologics like omalizumab—characterized by large size, intricate higher-order structures, heterogeneous post-translational modifications (PTMs) such as glycosylation, free thiol variants and the presence of multiple product-related variants—poses significant challenges for manufacturing consistency [9,10,11].

CMAB007 (Aomaishu^®^) is the first biosimilar of omalizumab approved in China in May 2023 [12,13,14]. To enhance production efficiency and the sustainability of post-approval, specific process optimizations were implemented. These included upstream media optimization and the replacement of hydrophobic interaction chromatography (HIC) with anion-exchange chromatography (AEX) in downstream purification. These changes achieved a 5-fold increase in production yield while retaining the original production cell line, aligning with the principles of Quality by Design (QbD) [15].

Global regulatory frameworks, established by authorities such as the International Council for Harmonization of Technical Requirements for Pharmaceuticals for Human Use (ICH), World Health Organization (WHO), the U.S. Food and Drug Administration (FDA), the European Medicines Agency (EMA) and center for drug evaluation of China (CDE) [16,17,18,19,20,21,22,23], provide structured guidance for assessing manufacturing changes for biologics, emphasizing risk-based comparability exercises. Industry practices for managing post-approval changes in biosimilars have also been documented in recent case studies [24,25,26]. However, the application of these guidelines to post-approval changes for biosimilars presents a unique challenge. Relying solely on conventional chemical drug change protocols or standard biosimilar development comparability approaches may be insufficient to mitigate the risk of “biological drift”—a subtle but consequential shift in critical quality attributes (CQAs) over time, which has been documented even for originator products [27,28,29,30,31]. This risk is amplified when a biosimilar, already demonstrated to be highly similar to the reference product, undergoes its own process changes. A strategy is needed that not only bridges pre- and post-change biosimilars but also continuously verifies alignment with the reference product’s quality profile.

To address this challenge, we pioneered a novel, integrated three-arm comparability strategy. This approach systematically compared the post-change product not only against the reference product (Xolair^®^) but also against its pre-change counterpart. Quality attributes were evaluated using a tiered, risk-based assessment with attribute-specific acceptance criteria. The comprehensive analytical characterization encompassed primary and higher-order structures, PTMs, purity/impurities, bioactivity, and Fc receptor-mediated functions, supplemented by forced degradation and accelerated stability studies. To clinically validate the strategy and confirm the absence of impactful drift, a pharmacokinetic (PK) comparability study comparing the post-change CMAB007 with the reference product was conducted in healthy male subjects.

This report describes the successful application of this tiered three-arm strategy, establishing a robust paradigm for managing post-approval manufacturing changes for biosimilars. The approach ensures the ongoing quality, efficacy, and safety of the biosimilar while facilitating necessary process improvements for scalable and sustainable production.

## 2. Results

### 2.1. Process Optimization Achieved a 5-Fold Yield Increase Without Altering Cell Line

**Figure 1:**
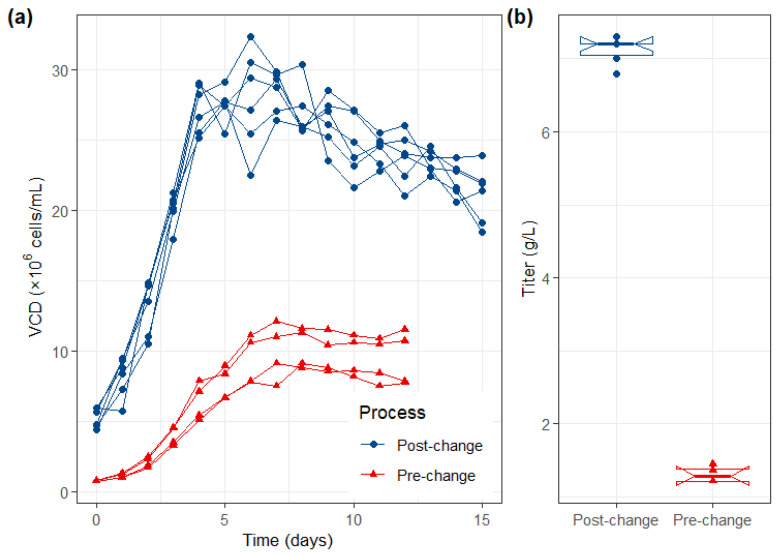
Enhanced production performance following process optimization. (**a**) Viable Cell Density (VCD) and (**b**) titer of pre-change and post-change product. Red line: pre-change product; blue line: post-change product.

The post-approval process optimization for CMAB007 successfully enhanced production efficiency while maintaining the original host cell line (007/B4-C2WCB). Key modifications included upstream media optimization and the replacement of hydrophobic chromatography with anion chromatography in the downstream process. These changes (Table 1), coupled with an extended culture duration (from 10–14 days to 12–16 days), resulted in a consistent and robust 5-fold increase in protein yield (Figure 1). Critically, the post-change intermediates demonstrated comparable impurity clearance and quality attributes to the pre-change material, confirming that the optimization did not adversely affect the basic product profile.

### 2.2. Three-Arm Tiered Analytical Comparison Confirms Quality Comparability

A novel three-arm comparability strategy, comparing the post-change product against both the pre-change product and the reference product (Xolair^®^), was implemented. QAs were classified into a three-tier system based on risk assessment, with tier-specific acceptance criteria (Table 2). This approach ensured a rigorous, risk-based evaluation.

#### 2.2.1. Physicochemical Properties

Comprehensive characterization confirmed that the post-change product was highly similar to both the pre-change and reference products (Table S1). The primary structure, including amino acid sequence and disulfide bond linkages, was identical. PTMs, such as glycosylation at the conserved site (EEQYNSTYR peptide), were consistent. Minor, non-critical differences were observed, such as a slightly higher level of fucosylated biantennary oligosaccharides in the post-change product compared to the reference, but this value was within the range of the pre-change product and is not considered a CQA for omalizumab due to its Fc-independent mechanism of action [32,33] (Table S1). Similarly, C-terminal lysine truncation was lower in CMAB007 (both pre- and post-change) compared to the reference, a difference with no known clinical impact [34,35,36,37,38,39]. Higher-order structure analyses (DSC, CD, DLS, and intrinsic fluorescence) confirmed conformational similarity across all three arms.

#### 2.2.2. Bioactivity and Immunological Characteristics

**Figure 2:**
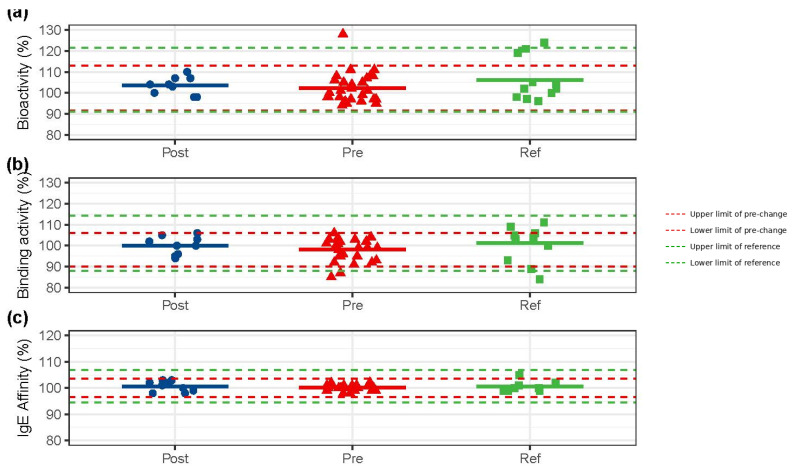
Functional equivalence demonstrated by Tier 1 quality attributes. (**a**) Bioactivity, (**b**) binding activity and (**c**) IgE affinity of post-change, pre-change and reference products. Red dashed lines: upper and lower limits of pre-change QRs (bioactivity: 91–113%; binding activity: 90–106%; IgE affinity: 97–104%); green dashed lines: upper and lower limits of reference QRs (bioactivity: 91–121%; binding activity: 88–114%; IgE affinity: 94–107%).

The functional integrity of the post-change product was thoroughly demonstrated. Tier 1 attributes, bioactivity and IgE binding activity, for the post-change product were within the pre-defined acceptance ranges (pre-change/reference mean ± 1.5 SD) of both the pre-change and reference products. Affinity to IgE, a Tier 2 attribute, was also equivalent. Furthermore, SPR analysis confirmed comparable affinity to a comprehensive panel of Fcγ receptors (FcγRI, FcγRIIa, FcγRIIb, FcγRIIIa, and FcγRIIIb), FcRn, and C1q, aligning the post-change product’s immunological characteristics with both the pre-change and reference profiles (Figure 2, Table S1).

#### 2.2.3. Purity and Impurity Profiles

**Figure 3:**
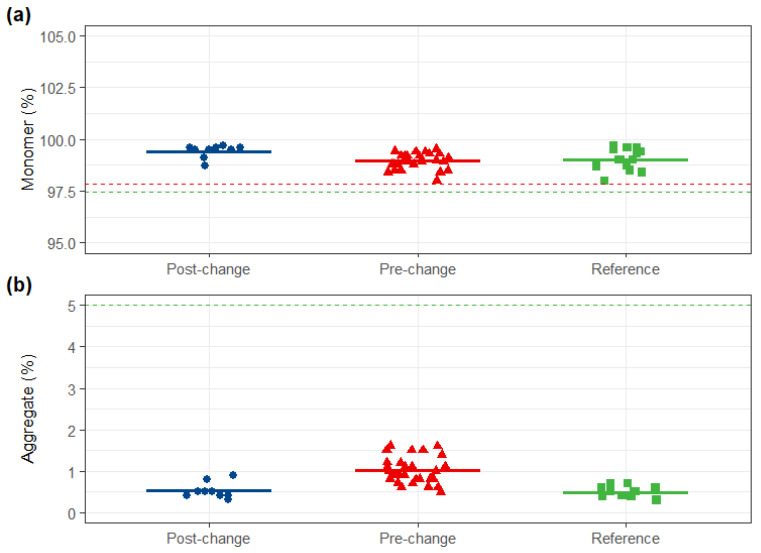
SEC-HPLC comparison. (**a**) Monomer; (**b**) aggregate of post-change, pre-change and reference products. Red dashed lines: upper and lower limits of pre-change QRs (monomer: ≥97.8%; aggregate: ≤5.0%); green dashed lines: upper and lower limits of reference QRs (monomer: ≥97.5%; aggregate: ≤5.0%).

**Figure 4:**
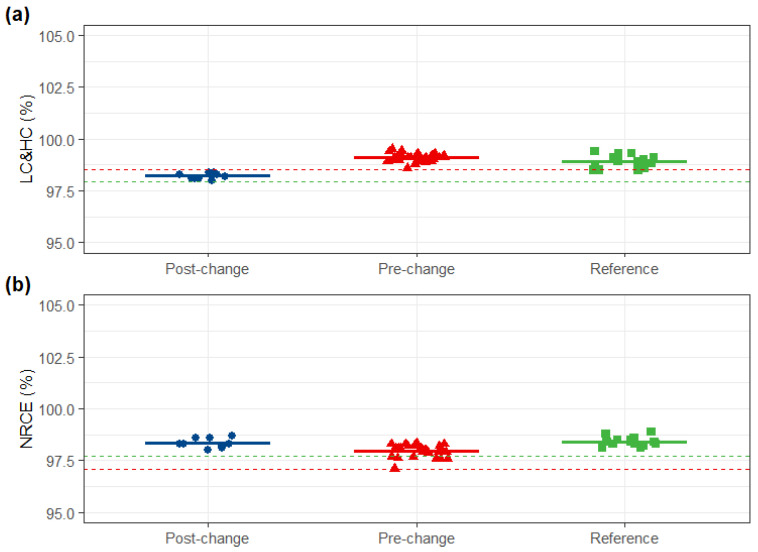
CE-SDS comparison. (**a**) LC&HC (**b**) NRCE of post-change, pre-change and reference products. Red dashed lines: lower limits of pre-change QRs (LC&HC: ≥98.5%; NRCE: ≤97.1%); green dashed lines: lower limits of reference QRs (LC&HC: ≥98.0%; NRCE: ≤97.7%).

**Figure 5:**
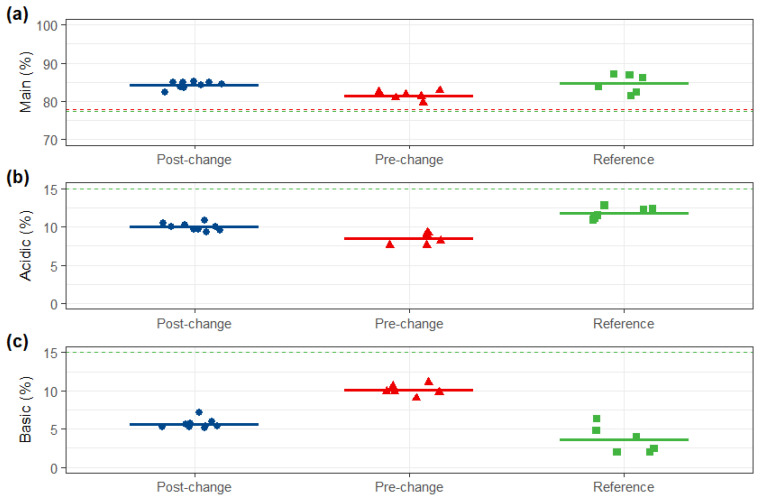
IEX-HPLC comparison. (**a**) Main, (**b**) acidic, and (**c**) basic of post-change, pre-change and reference products. Red dashed lines: upper and lower limits of pre-change QRs (main: ≥78.0%; acid: ≤15.0%; basic: ≤15.0%); green dashed lines: upper and lower limits of reference QRs (main: ≥77.4%; acid: ≤15.0%; basic: ≤15.0%).

**Figure 6:**
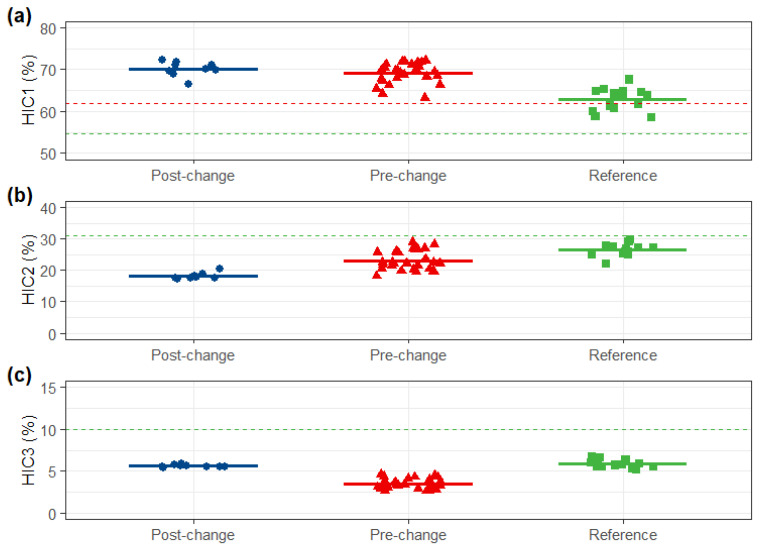
HIC-HPLC comparison. (**a**) HIC1, (**b**) HIC2 and (**c**) HIC3 of post-change, pre-change and reference products. Red dashed lines: upper and lower limits of pre-change QRs (HIC1: ≥61.9%; HIC2: ≤31.0%; HIC3: ≤10.0%); green dashed lines: upper and lower limits of reference QRs (HIC1: ≥54.7%; HIC2: ≤31.0%; HIC3: ≤10.0%).

The purity and variant distribution of the post-change product were highly similar to both comparators. SEC-UPLC revealed that monomer and aggregate content met the reference and the pre-change similarity standards. Both reduced and non-reduced capillary electrophoresis (CE-SDS) were highly similar across all three groups. Furthermore, charge variant profiles (IEX-UPLC) and hydrophobic variant profiles (HIC-UPLC) of the post-change product were consistent with those of the pre-change product and within the reference variability (Figure 3, Figure 4, Figure 5 and Figure 6).

Isolation and characterization of key variants (e.g., aggregates, acidic/basic peaks, HIC peaks, and non-glycosylated heavy chains) confirmed that they were present in all three arms and exhibited comparable bioactivity and binding activity, indicating a lack of functional impact from the process change (Table 3 and Table 4) [40,41]. This functional similarity indicates that the process changes did not alter the product’s critical quality attributes.

### 2.3. Stability Profile

Forced degradation and accelerated stability studies demonstrated that the post-change product exhibited comparable or improved stability relative to the pre-change product, with profiles closely aligned to the reference (Tables S2–S5).

Under accelerated conditions (25 °C), the increase in aggregates for the post-change product (0.3–0.7%) was lower than that of the pre-change product and similar to the reference (Table S2). Under more strenuous stress conditions (high temperature, photolysis, and post-reconstitution oxidation), the post-change product consistently showed reduced degradation rates compared to the pre-change product, as measured by key indicators like aggregate formation (SEC) and variant shifts (IEX and HIC). Its degradation profile under these stresses was highly similar to that of the reference product (Xolair^®^) (Tables S3–S5). These data confirmed the robustness of the modified manufacturing process.

### 2.4. Clinical PK Study Establishes Equivalence Between Post-Change and Reference Products

A randomized, double-blind, parallel-controlled clinical trial (registration number: CTR20240714, *n* = 114 healthy males) established the pharmacokinetic (PK) equivalence of the post-change CMAB007 to the reference product.

**Figure 7:**
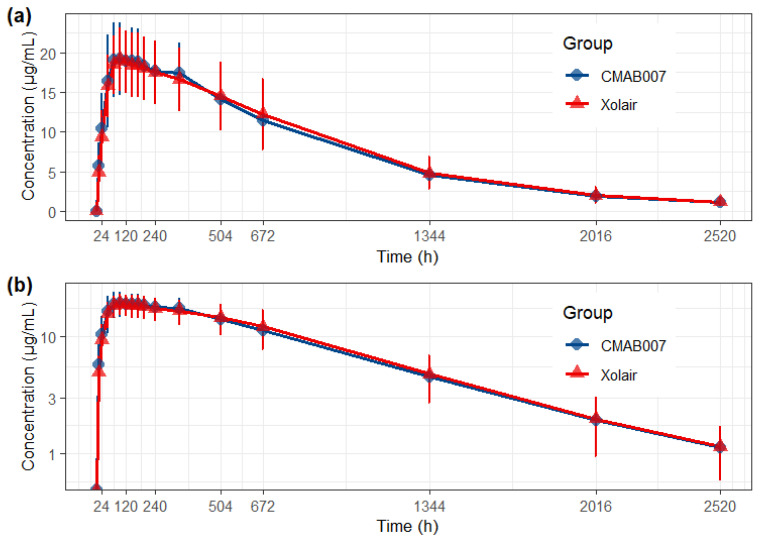
PK equivalence of post-change CMAB007 and reference product. Mean (±SD) serum concentration–time profiles following a single 150 mg subcutaneous dose of post-change CMAB007 (test) and Xolair^®^ (reference) in healthy male subjects. (**a**) Linear scale; (**b**) semilogarithmic scale.

Following a single 150 mg subcutaneous dose, the serum concentration–time profiles were nearly superimposable (Figure 7). Statistical analysis confirmed PK equivalence, as the 90% CI for the geometric mean ratios (GMRs) of all primary PK endpoints were entirely within the pre-specified 80.00–125.00% bioequivalence margin:

(1) AUC_0–inf_: GMR 99.82% (90% CI: 91.46~108.94%);
(2) AUC_0–t_: GMR 99.54% (90% CI: 91.40~108.41%);
(3) C_max_: GMR 101.88% (90% CI: 95.21~109.01%).

**Figure 8:**
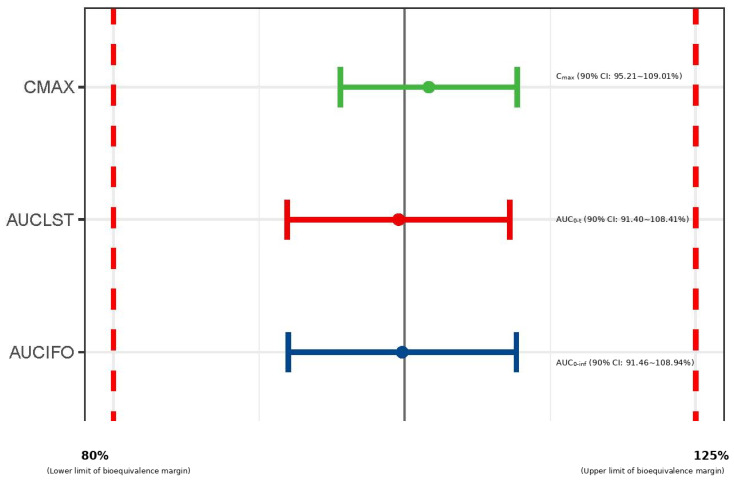
Statistical demonstration of PK bioequivalence. Green line: C_max_ (90% CI: 95.21~109.01%); red line: AUC_0–t_ (90% CI: 91.40~108.41%); blue line: AUC_0–inf_ (90% CI: 91.46~108.94%). The red dashed lines indicate the upper and lower limits of bioequivalence margin.

These results demonstrate that the absorption, distribution, and elimination of the post-change CMAB007 were equivalent to the originator, omalizumab (Table 5, Figure 8).

### 2.5. Comparable Safety and Immunogenicity Profiles

The safety and immunogenicity of the post-change product were comparable to the reference product.

The incidence of treatment-emergent adverse events (TEAEs) was 50.9% (29/57) in the CMAB007 group and 57.1% (32/56) in the Xolair^®^ group. Treatment-related adverse events (TRAEs) occurred in 24.6% (14/57) and 23.2% (13/56) of subjects, respectively. Notably, significant TEAEs and injection-site reactions were only reported in the reference group (1.8%, 1/56). No TEAEs of CTCAE Grade ≥ 3, allergic reactions, treatment discontinuations, special-interest AEs, serious adverse events (TESAEs), or fatal events occurred in either group.

Immunogenicity incidence was low and similar between groups. The proportion of subjects with at least one positive ADA result was 10.5% (6/57) for CMAB007 and 12.5% (7/56) for Xolair^®^ (*p* = 0.742). No Nabs were detected in any subject throughout the study duration.

## 3. Discussion

This study successfully demonstrates the application of a novel, integrated three-arm quality comparability strategy to manage post-approval process changes for an omalizumab biosimilar, CMAB007. The primary objective was to ensure that significant process enhancements—which yielded a 5-fold increase in production—did not adversely impact the critical quality, efficacy, or safety profile of the product. The strategy’s core innovation lies in its systematic, three-way comparison (post-change vs. pre-change vs. reference) coupled with a tiered, risk-based analytical assessment, providing a robust framework to proactively mitigate the risk of “biological drift” [28].

Our approach addresses a critical gap in the current paradigm for managing post-approval changes in biosimilars. While regulatory guidelines (e.g., ICH Q5E) provide a general framework for comparability exercises, and the tiered approach is well-established for initial biosimilar development [16,17,18,19,20,21,22], their combined application in a three-arm design for a post-approval scenario is pioneering. This strategy is particularly powerful for biosimilars because it simultaneously verifies two key relationships: first, that the post-change product remains comparable to its pre-change self (ensuring process control and consistency), and second, that both the pre- and post-change products remain aligned with the reference product’s quality range (safeguarding against drift from the originator’s established profile) [42]. This dual verification is a more stringent requirement than a standard two-arm comparability study and offers a higher level of assurance for long-term product quality.

The comprehensive analytical data unequivocally confirmed the high similarity of the post-change CMAB007 to both the pre-change product and Xolair^®^ across a wide spectrum of attributes. The tiered system allowed for a scientifically rigorous and efficient assessment [43,44]. Tier 1 attributes, with the highest potential clinical impact (biological and binding activity), were controlled with the tightest criteria, and the post-change product comfortably met these standards. For Tier 2 and 3 attributes, any observed minor differences (e.g., in fucosylation levels or C-terminal Lys truncation) were not only within the pre-defined acceptance ranges but were also demonstrated to be consistent with the pre-change product’s profile and, critically, were justified as non-critical based on the known mechanism of action of omalizumab (e.g., Fc-independent) or existing scientific studies (e.g., rapid in vivo cleavage of C-terminal Lys) [33,45]. The enhanced stability profile of the post-change product under various stress conditions further underscores the robustness of the optimized process, suggesting potential improvements in product shelf-life and handling.

Given that CMAB007 is a biosimilar product, to avoid potential biological drift resulting from process changes, we selected the reference product rather than the pre-change product as the comparator in the PK comparability study. The clinical PK study served as the ultimate validation of this analytical comparability. The demonstration of PK equivalence between the post-change CMAB007 and the reference product in healthy subjects, with 90% CIs for all primary endpoints firmly within the 80–125% bioequivalence margin, provided direct evidence that the process changes did not alter the in vivo exposure profile [46]. This, combined with the comparable safety and immunogenicity results, translates the analytical similarity into clinical performance similarity, effectively closing the loop on the comparability exercise.

While the current nine-batch dataset demonstrates consistent product quality and supports the comparability conclusion, we recognize that ongoing process verification is essential for building comprehensive long-term consistent data. Continued process verification will be carried out on an ongoing basis. Additionally, as the study was conducted by the manufacturer, potential sponsor bias cannot be completely excluded; however, independent third-party analysis and regulatory oversight help mitigate this concern. In the clinical PK study, only healthy adult males (*N* = 114) were enrolled, excluding females, elderly adults, and patients with asthma or urticaria—the target populations for omalizumab. While this study design demonstrated the PK comparability of CMAB007 following process optimization, caution should be exercised when extrapolating these findings to broader populations. Continued monitoring of efficacy and safety in females, older adults, and the indicated patient populations is warranted in post-marketing surveillance and ongoing clinical studies.

In conclusion, the tiered three-arm strategy presented here establishes a new paradigm for managing post-approval changes for biosimilars. It moves beyond mere compliance to a more proactive, scientifically driven model that effectively de-risks manufacturing evolution. By integrating state-of-the-art analytics, a risk-proportionate assessment framework, and targeted clinical validation, this approach ensures the continuous quality, efficacy, and safety of a biosimilar throughout its lifecycle. This strategy not only supports the sustainable scalability and cost-effectiveness of biosimilar manufacturing but also provides a clear and rigorous roadmap for the biopharmaceutical industry and regulators navigating the challenges of post-approval process optimization.

## 4. Materials and Methods

### 4.1. Tested Samples

A comprehensive panel of batches was utilized for comparative analyses (Table 6):

(1) Pre-change CMAB007: 28 commercial-scale batches manufactured prior to process optimization (Taizhou Mabtech Pharmaceutical Co., Ltd., Taizhou, China).
(2) Post-change CMAB007: The 9 post-Change batches manufactured after process optimization (Taizhou Mabtech Pharmaceutical Co., Ltd.) were commercial-scale runs, and the acceptance criteria were based on the larger pre-change dataset (28 batches) and reference product (14 batches), in line with ICH Q5E guidance [16].
(3) Reference Product (Xolair^®^): 14 batches of the originator omalizumab (Novartis Europharm Limited, Dublin, Ireland), sourced from markets in China, the USA, and Europe to account for potential regional heterogeneity.

### 4.2. Quality Comparability Strategy

To rigorously assess the impact of process changes and mitigate the risk of “biological drift,” a novel three-arm comparability strategy was employed, comparing the post-change product directly against both the pre-change product and the reference product.

Quality attributes (QAs) with potential impact on a drug’s structure, mechanism of action (MoA), safety, and efficacy were identified (Table S1). A risk-ranking approach was applied, evaluating each QA based on its potential impact (on PK/PD, immunogenicity, safety, and efficacy) and the associated uncertainty regarding this impact. Based on this risk assessment, QAs were classified into tiers, adapted from established biosimilar development principles [47,48].

(1) Tier 1 (High-Risk Impact): For QAs with a known high impact on clinical outcomes (e.g., bioactivity). Acceptance criteria: Post-change values must fall within the pre-change/reference product’s mean ± 1.5 standard deviations (SDs).
(2) Tier 2 (Medium-Risk Impact): For QAs with a potential or quantifiable impact (e.g., glycan profiles and key impurities). Acceptance criteria: Post-change values must fall within the pre-change/reference product’s mean ± 3 SDs.
(3) Tier 3 (Low-Risk Impact): For QAs with lower risk or those assessed qualitatively (e.g., a primary structure or higher-order structure). Acceptance was based on matching theoretical values or demonstrating qualitative similarity in profiles, trends, or magnitudes.

### 4.3. Analytical Characterization

A comprehensive suite of state-of-the-art analytical techniques was deployed to assess a wide spectrum of quality attributes.

#### 4.3.1. Physicochemical Properties

Intact molecular weight analysis: Intact and deglycosylated molecular weights were determined by liquid chromatography–mass spectrometry (LC-MS). After separation from buffer components, samples were analyzed on a Waters BEH300 C4 column (2.1 × 50 mm, 1.7 μm). Mass spectrometric detection was performed on a Waters Xevo G2-S system (Waters Corporation, Milford, MA, USA) operated in positive ion mode with an injection amount of 0.5 μg. The scan time was set to 0.5 s, the desolvation and source temperatures were 450 °C and 150 °C, respectively, the capillary and cone voltages were 3000 V and 110 V, the desolvation and cone gas flows were 800 and 50 L/h, respectively, and the mass range was 500–4000 *m*/*z*.

Peptide mapping: LC-MS peptide mapping was used to confirm the amino acid sequence, identify CDR-containing peptides, and characterize product-related post-translational modifications (PTMs), including glycosylation, oxidation, and deamidation [13,49]. Relative abundances of PTMs were calculated using Waters UNIFY software (version 3.12.0.548). Peak intensities in the deconvoluted spectra were normalized to the total signal, and relative percentages were derived from peak-height ratios.

Disulfide bond mapping: Disulfide bond linkages were characterized by LC-MS to verify the expected interchain and intrachain disulfide connectivity and to support the assessment of primary structural integrity [13,49].

High-order structures (HOSs): Conformational integrity was assessed by circular dichroism (CD; Chirascan Plus V100, Applied Photophysics, Leatherhead, UK), differential scanning calorimetry (DSC; Malvern VP-Capillary DSC Platform, Malvern Panalytical, Malvern, UK) for thermal transition profiles [50,51], dynamic light scattering (DLS; Malvern Zetasizer Nano ZS90, Malvern Panalytical, Malvern, UK) for particle size distribution and aggregation, and intrinsic fluorescence spectroscopy using a SpectraMax M5 microplate reader (Molecular Devices, San Jose, CA, USA) [52,53,54].

#### 4.3.2. Bioactivity and Immunological Characteristics

Functional Assays: Bioactivity [enzyme-linked immunosorbent assay (ELISA)]—FcεR was coated onto a 96-well plate, followed by adding a mixture of CMAB007 and IgE-HRP. Binding was detected using IgE-HRP followed by the 3,3′,5,5′-tetramethylbenzidine (TMB) reagent. Binding activity (ELISA)-IgE was coated onto a 96-well plate, followed by adding CMAB007. Binding was detected using Goat Anti-Human Kappa-HRP followed by the TMB reagent. Both assays were determined using validated ELISA methods.

Binding Affinity: The binding affinity to human IgE and a panel of Fcγ receptors (FcγRI, FcγRIIa, FcγRIIb, FcγRIIIa, and FcγRIIIb) and the neonatal Fc receptor (FcRn) was quantified using surface plasmon resonance (SPR; Biacore, Cytiva, Uppsala, Sweden). Binding to C1q was assessed by an ELISA.

#### 4.3.3. Purity and Impurities

Variant analysis: Purity and product-related variants were characterized using size-exclusion chromatography (SEC-UPLC) for aggregates and fragments, ion-exchange chromatography (IEX-UPLC) for charge variants, and HIC-UPLC for hydrophobic variants. Purity and size heterogeneity under denaturing conditions were analyzed by reduced and non-reduced capillary electrophoresis–sodium dodecyl sulfate (CE-SDS) [9,35,55].

SEC-UPLC measurements were performed on a Waters H-class UPLC system with a Waters ACQUITY UPLC^®^ Protein BEH SEC 200 Å column (1.7 μm 4.6 × 150 mm column). The SEC-UPLC system was equipped with a TUV or PDA detector (Waters Corporation, Milford, MA, USA). Samples were diluted to 10 mg/mL and then injected into the UPLC system. Elution was performed using 200 mM sodium phosphate (pH 7.0) at a flow rate of 0.2 mL/min, monitored by UV absorbance at 280 nm. HMW species and monomers were calculated based on the ratio of its peak area to the sum of the peak areas of all components in the chromatogram.

IEX-UPLC measurements were performed on a Waters H-class UPLC system with a Pro Pac^®^ SCX-10 analytical column, 4.0 × 150 mm column (Thermo Fisher Scientific, Waltham, MA, USA), eluted using a salt gradient and monitored by UV absorbance at 280 nm. Purity was evaluated by determining the peak area of each charged isoform group (main, acidic, and basic) that eluted separately as a percentage of the total peak area.

HIC-UPLC measurements were separated on a Waters e2695 Alliance HPLC system (Waters Corporation, Milford, MA, USA) with a TSK Phenyl-5PW 7.5 × 75 mm (TOSOH, Tokyo, Japan), eluted using a salt gradient and monitored by UV absorbance at 214 nm. Purity was evaluated by determining the peak area of each charged isoform group that eluted separately as a percentage of the total peak area.

For non-reduced CE-SDS (nrCE-SDS) and reduced CE-SDS (rCE-SDS), samples were prepared using the ProteinSimple Maurice (ProteinSimple, San Jose, CA, USA). For reduced CE-SDS (rCE-SDS), 2-mercaptoethanol (Sigma-Aldrich, St. Louis, MO, USA) was added to the protein denaturation (100 mMTris pH 9.0, 1% SDS) step to reduce the disulfide bonds. After denaturation, both non-reduced CE-SDS (nrCE-SDS) and rCE-SDS samples were injected onto a bare, fused silica capillary (AB Sciex, Framingham, MA, USA) and separated based on hydrodynamic size resulting from an applied electric field in which migration of smaller-sized proteins is inversely related to overall size. Analytes were monitored by UV absorbance at 220 nm, and purity was evaluated by determining the peak area of each species as a percentage of the total peak area.

Variant Identification: Critical variant peaks (size, charge, and hydrophobic) were isolated and collected. Their structures were identified using LC-MS, and their biological and binding activities were functionally characterized to assess potential impact.

### 4.4. Stability Studies

The comparative stability of the post-change, pre-change, and reference products was evaluated under stress conditions to assess structural robustness and degradation pathways.

Stability-indicating methods included SEC-UPLC (Waters UPLC H-Class, Waters Corporation, Milford, MA, USA), IEX-UPLC (Waters UPLC H-Class), HIC-UPLC (Waters e2695 Alliance HPLC, Waters Corporation, Milford, MA, USA), CE-SDS (ProteinSimple Maurice, ProteinSimple, San Jose, CA, USA), and assessments of bioactivity (SpectraMax 190, Molecular Devices, San Jose, CA, USA) and binding activity (SpectraMax 190, Molecular Devices, San Jose, CA, USA).

The selection of stability-indicating methods was based on ICH Q5C guidelines, which state that methods contributing to stability assessment include electrophoresis (SDS-PAGE, immunoelectrophoresis, Western blot, and isoelectrofocusing), high-resolution chromatography (e.g., reversed-phase chromatography, gel filtration, ion exchange, and affinity chromatography), and peptide mapping [56].

Consistent with precedent in biosimilar stability studies [24,25,26], we selected SEC-UPLC, IEX-UPLC, HIC-UPLC, and CE-SDS to monitor purity and molecular characteristics, complemented by bioactivity and binding activity assessments. Additionally, the accelerated and long-term stability studies for pre- and post-change CMAB007 included cIEF, peptide mapping, color, dissolution time for powders, visible particulates after reconstitution, pH, moisture, and sterility testing.

#### 4.4.1. Accelerated Stability

Products were stored at 25 °C ± 2 °C for up to 6 months.

#### 4.4.2. Forced Degradation

Studies included (Table 7):

High-temperature stress: 40 °C ± 2 °C for up to 30 days.

Photostability: Exposure to overall illumination ≥ 1.2 million lux hours and integrated near-ultraviolet energy of ≥200 watt hours/square meter.

Oxidative Stress (post-reconstitution): Exposure to 3% H_2_O_2_ at 5 °C ± 3 °C for up to 2 days.

### 4.5. Clinical Bridging Study

To confirm the pharmacokinetic (PK) and safety comparability of the post-change product relative to the reference product, a clinical study was conducted. The pre-change product was not included in this clinical study.

#### 4.5.1. Study Design

A randomized, double-blind, parallel-controlled trial was performed (Clinical Trial Registry number: CTR20240714).

The PK study utilized single-dose administration rather than repeated dosing based on the following considerations: (a) single-dose PK parameters (AUC_0–∞_, C_max_) are most sensitive for detecting formulation-related bioavailability differences between biosimilar and reference products; (b) omalizumab’s long elimination half-life (~26 days) makes multiple-dose studies impractical for initial comparability assessment; and (c) immunogenicity evaluation in a single-dose study provides initial safety signals, with comprehensive immunogenicity data obtained from the multiple-dose Phase 3 studies of CMAB007 [57]. This approach is consistent with established biosimilar development paradigms.

#### 4.5.2. Participants

A total of 114 healthy adult male subjects were enrolled and randomized to receive a single 150 mg subcutaneous dose of either post-change CMAB007 (*n* = 57) or Xolair^®^ (*n* = 57).

#### 4.5.3. PK Sampling and Endpoints

Blood sampling was conducted over 106 days (covering > 5 half-lives) to fully characterize the PK profile. Primary endpoints were the area under the concentration–time curve from zero to infinity (AUC_0–inf_) and maximum observed concentration (C_max_). Secondary endpoints included the AUC from zero to the last measurable concentration (AUC_0-–t_), time to C_max_ (T_max_), elimination half-life (t_1/2_), apparent clearance (CL/F), and apparent volume of distribution (Vd/F).

#### 4.5.4. Safety and Immunogenicity

Safety was assessed via adverse events (AEs), vital signs, laboratory parameters, and electrocardiograms. Immunogenicity was evaluated by measuring the incidence of anti-drug antibodies (ADAs) and neutralizing antibodies (NAbs) at multiple timepoints.

#### 4.5.5. Statistical Analysis

PK parameters were log-transformed and analyzed using an analysis of variance (ANOVA) model. PK comparability was concluded if the 90% confidence intervals (CIs) for the geometric mean ratios (Test/Reference) of AUC_0–inf_, AUC_0-t_, and C_max_ were entirely within the pre-defined equivalence margin of 80.00% to 125.00%. Statistical analyses were performed using WinNonlin^®^ (version 8.4) and SAS^®^ (version 9.4).

## Figures and Tables

**Table 1:** Summary of key manufacturing process changes.

Parameter	Post-Change	Pre-Change	Comparison
Cell line	007/B4-C2WCB	007/B4-C2WCB	Identical
Bioreactor scale	1500 L	1500 L	Identical
Medium	Basal media: CHOM-B03; CHOM-B02; Supplement media: CHOM-S03; CHOM-S04	Basal media: CHOM-B02; Supplement media: CHOM-S01	Chemically defined media; Same supplier
Culture duration	12–16 days	10~14 days	Extended for higher yield
Purification	Affinity + cation/anion	Affinity + cation/hydrophobic	Hydrophobic to anion

**Table 2:** Tier classification and acceptance criteria for critical quality attributes.

Tier	Quality Attributes	Acceptance Criteria
Tier1	Binding activity and bioactivity	Pre-change/reference mean ± 1.5 SD
Tier2	Hexose content, glycosylation profiles (fucosylated biantennary oligosaccharides), IgE/FcγRIIIa/FcRn affinity, NR-CE, R-CE, SEC-UPLC, IEX-UPLC, HIC-UPLC	Pre-change/reference mean ± 3 SD *
Tier3	Amino acid sequence, molecular weight, primary structure (subunit analysis), disulfide bonds,	Matches theoretical values or identical
	free thiols, glycation, PTMs, DSC, CD spectroscopy, intrinsic fluorescence, peptide mapping, cIEF, particle size distribution, FcγRI/FcγRIIa/FcγRIIb/FcγRIIIb/C1q affinity, stability studies (forced degradation, accelerated stability)	Qualitative comparisons (profile similarity, limits, magnitude, trends)

* For impurities, similarity acceptance criteria were set as fixed specification limits.

**Table 3:** Structural identification of charge variants by LC-MS following carboxypeptidase B (CPB) digestion.

IEX	Post-Change	Pre-Change	Reference
Acidic	SA, Deamidation, Glycation, Oxidation	SA, Deamidation, Glycation, Oxidation	SA, Deamidation, Glycation, Oxidation
Main	0 K	0 K	0 K
Basic	PyrE	PyrE	PyrE

PyrE: N-terminal pyroglutamate cyclization; 0 K: complete C-terminal lysine truncation; SA: sialic acid modification.

**Table 4:** Bioactivity and binding activity of charge variants.

IEX	Bioactivity			Binding Activity		
	Post-Change	Pre-Change	Reference	Post-Change	Pre-Change	Reference
Acidic	93–102%	120%	117%	83–97%	99%	99%
Main	93–109%	126%	117%	87–109%	106%	118%
Basic	89–97%	121%	115%	83–104%	83%	101%

**Table 5:** Summary of PK parameters following a single 150 mg subcutaneous dose.

Parameter	Post-Change CMAB007 Group	Xolair^®^ Group
C_max_	21.23 μg/mL (21.23% CV)	20.83 μg/mL (21.15% CV)
T_max_	120.00 h (47.99–337.27 h)	120.00 h (47.99–550.90 h)
AUC_0–t_	18,158.89 h·μg/mL (23.60% CV)	18,526.98 h·μg/mL (30.68% CV)
AUC_0–inf_	19,046.76 h·μg/mL (24.49% CV)	19,380.29 h·μg/mL (31.64% CV)
t_1/2_	528.60 h (17.46% CV)	506.07 h (14.67% CV)
Vd/F	6208.36 mL (18.49% CV)	6015.42 mL (22.86% CV)
CL/F	8.39 mL/h (26.51% CV)	8.48 mL/h (30.16% CV)

**Table 6:** Product batches used for comparative studies.

Sample	Batches for Quality Comparison	Batches for Stability	Batches for PK Study
Pre-change CMAB007	28	3	/
Post-change CMAB007	9	3	1
4Reference (Xolair^®^)	14	3	1

**Table 7:** Conditions for accelerated and forced degradation stability studies.

Study		Conditions	Timepoints	Tests
Accelerated stability		25 °C ± 2 °C	0, 1, 2, 3, 6 months	NR-CE, R-CE, SEC/IEX/HIC-UPLC, bioactivity, binding activity
Forced degradation	High temperature	40 °C ± 2 °C	0, 10, 30 days	NR-CE, R-CE, SEC/IEX/HIC-UPLC, bioactivity, binding activity
	Photostability	Overall illumination ≥ 1.2 million lux hours and an integrated near-ultraviolet energy ≥ 200 watt hours/square meter	0, 11, 30 days	As above
	Oxidation (post-reconstitution)	3% H_2_O_2_, 5 °C ± 3 °C	0, 1, 2 days	As above

## Data Availability

The original contributions presented in the study are included in the article and Supplementary Materials, further inquiries can be directed to the corresponding authors.

## Supplementary Materials

The following supporting information can be downloaded at: https://www.mdpi.com/article/10.3390/ph19050724/s1, Figure S1: Representative chromatograms/spectra; Figure S2: Stability Data from stability studies (Accelerated testing); Figure S3: Stability Data from stability studies [High-temperature testing (40 °C)]; Figure S4: Stability Data from stability studies (Photostability testing); Figure S5: Stability Data from stability studies [Oxidative stress (post-reconstitution)]; Table S1: Quality comparison results; Table S2: Accelerated testing for pre-change, post-change and reference; Table S3: Forced degradation studies under high temperature(40 °C) for pre-change, post-change and reference; Table S4: Forced degradation studies under photostability testing for pre-change, post-change and reference; Table S5: Forced degradation studies under oxidative stress (post-reconstitution stability) for pre-change, post-change and reference.
