# Supplementary material for: A Three-Arm, Tiered Comparability Strategy Bridging Post-Approval Process Changes for an Omalizumab Biosimilar (CMAB007)
Source: Pharmaceuticals (Basel). 2026 May 2;19(5):724. doi: 10.3390/ph19050724 (PMC13209631; doi:10.3390/ph19050724)
Supplement: Supplementary file 1 [file pharmaceuticals-19-00724-s001.zip › pharmaceuticals-4221418-supplementary.pdf]

A Three-Arm, Tiered Comparability Strategy Bridging Post-Approval Process Changes for an Omalizumab Biosimilar (CMAB007)

Supplementary Material

Chenguang Wang[1,2,3,4,†], Chaoxin Zhou[1,2,3,4,†],Sheng Hou[1,2,3,4], Wenqiang Fan[1], Weizhu Qian[1,2,3,4], Yule Ren[1,2,3,4], Xiyuan Chen[4], Chenhong Pan[4], Qingcheng Guo[1,2,3,4], Huaizu Guo[1,2,3,*], Yajun Guo[1,2,3,*]

[1]State Key Laboratory of Macromolecular Drugs and Large-scale Preparation, School of Pharmaceutical Sciences, Wenzhou Medical University, Wenzhou, China

[2]State Key Laboratory of Macromolecular Drugs and Large-scale Preparation, NMPA Key Laboratory for Quality Control of Therapeutic Monoclonal Antibodies, Shanghai Zhangjiang Biotechnology Co., Ltd, Shanghai, China

[3]State Key Laboratory of Macromolecular Drugs and Large-scale Preparation, School of Pharmaceutical Sciences and Food Engineering, Liaocheng University, Liaocheng, China.

[4]Taizhou Mabtech Pharmaceuticals Co., Ltd, Taizhou, China.

[†]These authors contributed equally to this work.

[*]Correspondence to: Huaizu Guo, guohuaizu@163.com and Yajun Guo, 18621716752@163.com. State Key Laboratory of Macromolecular Drugs and Large-scale Preparation, School of Pharmaceutical Sciences, Wenzhou Medical University, Wenzhou, China; State Key Laboratory of Macromolecular Drugs and Large-scale Preparation, School of Pharmaceutical Sciences and Food Engineering, Liaocheng University, Liaocheng, China.

List of Tables:

Table S1 Quality comparison results

Table S2. Accelerated testing for pre-change, post-change and reference

Table S3. Forced degradation studies under high temperature(40℃) for pre-change, post-change and reference

Table S4. Forced degradation studies under photostability testing for pre-change, post-change and reference

Table S5. Forced degradation studies under oxidative stress (post-reconstitution stability) for pre-change, post-change and reference

Figure S1. Representative chromatograms/spectra. (a) SEC-UPLC, (b) IEX-UPLC, (c) HIC-UPLC, (d) nrCE-SDS, (e) rCE-SDS, (f) bioactivity, (g) binding activity

Figure S2. Stability data from accelerated stability studies

Figure S3. Stability data from forced degradation under high-temperature stress (40°C)

Figure S4. Stability data from photostability testing

Figure S5. Stability data from oxidative stress testing

Table S1 Quality comparison results

Category		Test item		Tier	Post-change	Pre-change	Reference
Physicochemical properties	Primary structure and PTMs	Primary amino acid sequence	Peptide map	3	Light chain coverage rate: 100%, no different amino acid sequences or sequence variants were found		
				3	Heavy chain coverage rate: 100%, no different amino acid sequences or sequence variants were found, and the allotype sequence in the Fc region was confirmed		
				3	Peptide: consistent spectra		
				3	CDR region identification: the characteristic peptide fragments in the light chain CDR region and the heavy chain CDR region had been confirmed, and the retention time of peptide fragments in each CDR region was the same.		
				3	The N-terminal and C-terminal sequences of both the light and heavy chains were confirmed by MS/MS analysis. These sequences were consistent with the theoretical predictions, and the retention times of all peptide fragments were identical.		
		Molecular weight	LC-MS	3	Molecular weight of intact protein: consistent with the theoretical molecular weight, within an error of ± 100 ppm.		
				3	Intact protein peptide mapping: the peak profile and molecular weight were consistent with those of the reference, with only minor differences in the abundance of individual components, and no other significant differences		
			LC-MS (PNGase F treatment)	3	Molecular weight of deglycosylated intact protein: consistent with the theoretical molecular weight, within an error of ± 100 ppm.		
				3	Deglycosylated intact protein peptide mapping: consistent with the theoretical molecular weight		
		Primary structure and PTMs	LC-MS (subunit analysis)	3	Molecular weight of main peak of light chain: consistent with the theoretical molecular weight, within an error of ± 50 ppm		
				3	Post-translational modification of the light chain: The first amino acid was identified as Asp instead of Glu or Gln. Additionally, no N-terminal pyroglutamate was detected.		
				3	Molecular weight of heavy chain: consistent with the theoretical		

			molecular weight, within an error of ± 50 ppm		
		3	Post-translational modifications of the heavy chain: No significant N-terminal cleavage isoforms were observed.	Post-translational modifications of the heavy chain: No significant N-terminal cleavage isoforms were observed. The C-terminal Lys truncation rate of Xolair® was higher than CMAB007	
	LC-MS (subunit analysis, PNGase F treatment)	3	Molecular weight of light chain and heavy chain: consistent with the theoretical molecular weight, within an error of ± 50 ppm		
		3	Peptide mapping: the peak profile of light chain and heavy chain were consistent with those of the reference		
Disulfide bond	LC-MS/MS (Trypsin/Lys-C and Lys-C digestion)	3	Consistent with the theoretical disulfide bonds profile, and no mismatched disulfide bonds were detected		
Free thiol		3	0.270~0.548mol/mol	0.499~0.703mol/mol	0.602~0.831mol/mol
Glycosylation modification	LC-MS/MS	3	Modification sites: all occurred at conserved site. The secondary MS fragments of the glycosylated peptides were consistent. The glycosylated peptides were EEQYNSTYR, and the glycosylation occurred on the Asn of the peptides.		
		3	Glycosylation types: mainly G0F, G1F, and G2F		
		2	Fucosylated biantennary Oligosaccharides: 89.00~91.86%	Fucosylated biantennary Oligosaccharides: 86.88~92.01%	Fucosylated biantennary Oligosaccharides: 85.90~92.63%
		3	High mannose:＜10%		
		3	Sialic acid: NANA		
		3	Sialic acid＜ 11.7mmol/mol	Sialic acid＜ 19.63mmol/mol	Sialic acid＜ 19.33mmol/mol
		2	Hexose content: 6.74~8.27mol/mol	Hexose content: 7.22~12.01mol/mol	Hexose content: 7.05~10.73mol/mol
Other post-translational	LC-MS	3	N-terminal pyroglutamate:	N-terminal pyroglutamate:	N-terminal pyroglutamate:

	modifications			1.1~1.2%<br>C-terminal Lys truncation: 63.5~69.3%	1.1~3.2%<br>C-terminal Lys truncation: 76.3~87.6%	1.4~3.4%<br>C-terminal Lys truncation: 92.4~96.8%
			3	Oxidation: No confirmed oxidative modifications were observed in the CDR regions. The abundance of modified peptides was consistently low, with primary modification sites localized to the heavy chain constant region.		
			3	Deamidation: Deamidation occurred at consistent sites with similar proportions. The majority of deamidation was observed at classic sequences containing -NG/-QG motifs		
			3	Pyroglutamate: The heavy chain N-terminal amino acid was identified as Glu, with a minor proportion of pyroglutamate detected (<5%)		
			3	Glycation: sites and proportions were comparable		
High order structure	DSC		3	Tm1:70.24~70.97℃<br>Tm2: 82.01~82.40℃<br>Tm3: 86.91~87.06℃	Tm1: 68.83~69.31℃<br>Tm2: 80.70~81.99℃<br>Tm3: 86.62~86.97℃	Tm1:69.65~70.98℃<br>Tm2:81.32~82.09℃<br>Tm3:86.79~87.67℃
	DLS	Sizer	3	11.64~12.89d.nm	11.85~13.39d.nm	11.93~12.92d.nm
		Width		2.532~4.004d.nm	2.874~3.688d.nm	3.438~4.478d.nm
		Intensity		94.8~100.0%	97.1~100.0%	99.1~100.0%
	Intrinsic fluorescence		3	The spectrum was basically consistent		
	CD		3	The spectrum was basically consistent		
Bioactivity	Bioactivity		1	98~110%	94~128%	81~124%
	Binding activity		1	94~106%	85~104%	84~114%
	IgE affinity		2	98~103%	98~102%	98~105%
Purity and impurity	cIEF		3	the difference in the main peak pI was within ± 0.1<br>The spectrum was consistent		
	NR-CE		2	98.0~98.7%	97.1~98.3%	98.1~98.9%
	R-CE		2	98.0~98.4%	98.6~99.5%	98.5~99.4%
	SEC-monomer		2	98.7~99.7%	98.0~99.4%	98.0~99.7%
	SEC-aggregate		2	0.3~0.9%	0.7~1.6%	0.3~0.7%
	IEX-spectrum		3	The spectrum was consistent		
	IEX-main		2	82.3~85.3%	79.6~82.8%	77.1~80.5%

		IEX-basic		2	5.2~7.1%	9.0~11.1%	2.0~6.3%
		IEX-acidic		2	9.3~10.9%	7.6~9.2%	10.9~12.8%
		HIC-HIC1		2	66.5~72.3%	63.2~72.2%	58.5~67.6%
		HIC-HIC2		2	17.1~20.6%	18.2~29.0%	22.1~29.7%
		HIC-HIC3		2	5.4~5.9%	2.6~4.6%	5.2~6.7%
Immunological characteristics		Affinity with FcγRⅠ，CD64		3	101~102%	97~102%	97~103%
		Affinity with FcγRⅡa，CD32a		3	98~100%	82~125%	94~124%
		Affinity with FcγRⅡb，CD32b		3	99~102%	99~103%	99~106%
		Affinity with FcγRIIIa，CD16a		2	97~99%	98~115%	97~100%
		Affinity with FcγRIIIb，CD16b		3	98~99%	98~104%	93~104%
		Affinity with FcRn		2	96~103%	96~101%	97~105%
		Affinity with C1q		3	99~100%	99~102%	99~102%
Activity/characterization of product-related substances	SEC	Monomer	Bioactivity	3	105~110%	114%	97%
			Binding activity	3	97~111%	101%	98%
		Aggregate	identification		Formed through intermolecular non-covalent interactions		
			Bioactivity	3	95~104%	81%	92%
			Binding activity	3	75~78%	68%	75%
	IEX	Acidic	Bioactivity	3	98~102%	120%	117%
			Binding activity	3	83~97%	99%	99%
		Main	Bioactivity	3	93~109%	126%	117%
			Binding activity	3	87~109%	106%	118%
		Basic	Bioactivity	3	89~97%	121%	115%
			Binding activity	3	83~104%	83%	101%
	HIC	HIC2		3	Mainly modified by free thiol groups		
		HIC3		3	Mainly modified by aspartic acid isomers		

Table S2. Accelerated testing for pre-change, post-change and reference

Tests		Post-change	Pre-change	Reference
NR-CE	0	98.0~98.3%	97.9~98.1%	98.6~98.9%
	1 month	98.2~98.3%	96.8~97.2%	98.2~98.4%
	2 months	98.0~98.1%	96.4~96.9%	98.2~98.4%
	3 months	97.5~97.9%	96.3~96.9%	98.1~98.3%
R-CE	0	98.3~98.4%	99.0~99.1%	98.5~98.7%
	1 month	97.6~98.0%	98.5~98.7%	98.4~98.6%
	2 months	97.8~98.0%	98.3~98.5%	98.7~99.0%
	3 months	97.9~98.2%	97.6~98.0%	98.8~98.9%
SEC-monomer	0	98.7~99.5%	98.5~99.2%	99.6~99.7%
	1 month	99.1~99.2%	97.0~98.0%	99.3~99.3%
	2 months	98.9~99.0%	95.7~96.4%	99.1~99.1%
	3 months	98.7~98.8%	95.7~96.4%	98.9~99.0%
SEC-aggregate	0	0.5~0.9%	0.8~1.5%	0.3~0.4%
	1 month	0.8~0.9%	2.0~3.0%	0.7~0.7%
	2 months	1.0~1.1%	3.6~4.3%	0.8~0.9%
	3 months	1.2~1.3%	3.6~4.3%	1.0~1.0%
IEX-acidic	0	10.2~10.9%	NA*	10.9~11.5%
	1 month	9.9~10.2%		12.2~12.8%
	2 months	10.0~10.1%		12.3~12.7%
	3 months	9.8~10.2%		12.5~13.1%
IEX-main	0	82.3~83.8%		86.1~87.1%
	1 month	83.1~83.6%		80.3~80.6%
	2 months	82.9~83.5%		79.9~80.3%
	3 months	82.9~83.3%		79.6~81.6%
IEX-basic	0	5.6~7.1%		2.0~2.4%
	1 month	6.3~7.0%		6.9~7.3%
	2 months	6.5~7.0%		7.2~7.4%
	3	6.8~6.9%		5.9~7.3%

	months			
HIC-HIC1	0	70.0~72.3%	69.4~72.2%	60.8~61.7%
	1 month	72.0~72.8%	68.7~70.7%	54.5~64.7%
	2 months	70.3~71.5%	68.2~69.2%	61.9~63.4%
	3 months	70.6~71.7%	69.4~70.0%	56.7~57.6%
HIC-HIC2	0	17.1~18.0%	18.2~20.4%	27.1~27.9%
	1 month	17.6~18.5%	20.3~21.4%	27.2~27.9%
	2 months	16.7~17.4%	20.4~21.1%	25.4~26.1%
	3 months	17.8~18.9%	20.5~21.5%	29.4~30.1%
HIC-HIC3	0	5.6~5.9%	4.3~4.4%	5.3~5.5%
	1 month	4.8~4.9%	4.3~4.8%	4.0~4.2%
	2 months	6.1~6.3%	5.0~5.5%	5.8~6.1%
	3 months	5.3~5.4%	4.0~4.8%	5.8~6.0%
Bioactivity	0	103~107%	96~108%	97~102%
	1 month	94~109%	99~108%	96~98%
	2 months	99~107%	94~100%	93~94%
	3 months	102~109%	105~106%	96~101%
Binding activity	0	96~106%	95~103%	103~106%
	1 month	99~108%	97~100%	96~109%
	2 months	98~105%	100~103%	98~108%
	3 months	103~119%	100~108%	95~101%

NA: different IEX methods were used for pre-change

Table S3. Forced degradation studies under high temperature(40℃) for pre-change, post-change and reference

Tests		Post-change	Pre-change	Reference
NR-CE	0	98.0~98.3%	97.9~98.0%	98.6~98.9%
	10 days	97.7~97.9%	96.2~97.0%	98.0~98.2%
	30	97.3~97.8%	95.6~96.0%	97.8~97.9%

	days			
R-CE	0	98.3~98.4%	99.0~99.1%	98.5~98.7%
	10 days	97.7~97.9%	97.7~98.2%	98.5~98.6%
	30 days	97.7~7.8%	97.4~97.7%	98.2~98.8%
SEC-monomer	0	98.7~99.5%	98.8~99.3%	99.6~99.7%
	10 days	98.4~98.4%	95.3~96.9%	98.6~98.7%
	30 days	97.9~98.2%	95.2~95.6%	98.3~98.3%
SEC-aggregate	0	0.5~0.9%	0.7~1.2%	0.3~0.4%
	10 days	1.2~1.3%	2.8~4.4%	1.0~1.0%
	30 days	1.8~2.0%	4.4~4.8%	1.6~1.6%
IEX-acidic	0	10.2~10.9%	7.6~9.0%	10.9~11.5%
	10 days	9.9~10.0%	7.6~8.9%	12.2~12.8%
	30 days	10.3~10.4%	8.0~9.3%	12.7~13.2%
IEX-main	0	82.3~83.8%	81.2~82.4%	86.1~87.1%
	10 days	82.4~82.7%	77.1~78.9%	81.4~82.5%
	30 days	78.7~79.4%	69.9~72.5%	78.5~79.8%
IEX-basic	0	5.6~7.1%	9.8~10.6%	2.0~2.4%
	10 days	7.4~7.6%	13.5~15.3%	2.5~5.8%
	30 days	10.3~10.9%	19.5~20.8%	7.2~8.3%
HIC-HIC1	0	70.0~72.3%	70.3~72.2%	60.8~61.7%
	10 days	69.1~69.7%	65.7~67.6%	59.5~61.0%
	30 days	68.6~69.2%	63.8~66.4%	59.3~59.8%
HIC-HIC2	0	17.1~18.0%	18.2~23.6%	27.1~27.9%
	10 days	16.3~17.2%	19.2~21.1%	25.6~27.2%
	30 days	16.1~16.8%	18.2~20.4%	26.0~26.7%
HIC-HIC3	0	5.6~5.9%	3.7~4.4%	5.3~5.5%
	10 days	6.9~7.1%	5.8~6.3%	6.2~6.5%

	30 days	7.4~7.6%	7.8~8.1%	7.4~7.6%
Bioactivity	0	103~107%	96~102%	97~102%
	30 days	100~106%	97~105%	92~96%
Binding activity	0	96~106%	92~103%	103~106%
	30 days	90~109%	91~101%	96~99%

Table S4. Forced degradation studies under photostability testing for pre-change, post-change and reference

Tests		Post-change	Pre-change	Reference
NR-CE	0	98.0~98.3%	97.9~98.0%	98.6~98.9%
	11 days	96.1~96.6%	94.2~95.4%	97.0~97.3%
	30 days	93.2~94.4%	91.9~92.2%	94.8~95.2%
R-CE	0	98.3~98.4%	99.0~99.1%	98.5~98.7%
	11 days	97.8~98.1%	98.5~98.7%	98.5~98.8%
	30 days	97.6~97.8%	97.7~97.9%	98.2~98.3%
SEC-monomer	0	98.7~99.5%	98.8~99.3%	99.6~99.7%
	11 days	99.0~99.1%	96.9~97.7%	99.2~99.3%
	30 days	99.0~99.1%	96.7~97.8%	99.2~99.3%
SEC-aggregate	0	0.5~0.9%	0.7~1.2%	0.3~0.4%
	11 days	0.9~1.0%	2.3~3.1%	0.7~0.7%
	30 days	0.9~1.0%	2.2~3.2%	0.7~0.8%
IEX-acidic	0	10.2~10.9%	7.6~9.0%	10.9~11.5%
	11 days	12.2~12.9%	10.0~11.0%	13.9~14.4%
	30 days	14.5~15.7%	13.0~14.6%	15.8~16.5%
IEX-main	0	82.3~83.8%	81.2~82.4%	86.1~87.1%
	11 days	76.5~78.4%	73.3~74.8%	78.3~79.6%
	30 days	71.2~74.4%	67.3~69.8%	72.8~73.4%

IEX-basic	0	5.6~7.1%	9.8~10.6%	2.0~2.4%
	11 days	9.4~10.6%	14.2~16.3%	6.3~7.3%
	30 days	11.1~13.1%	17.1~18.1%	10.6~10.8%
HIC-HIC1	0	70.0~72.3%	70.3~72.2%	60.8~61.7%
	11 days	70.9~72.0%	69.1~71.1%	65.8~68.3%
	30 days	67.4~68.4%	65.0~67.4%	59.0~60.1%
HIC-HIC2	0	17.1~18.0%	18.2~23.6%	27.1~27.9%
	11 days	15.5~16.5%	18.0~20.1%	20.1~22.5%
	30 days	18.4~19.3%	20.8~22.9%	27.4~28.2%
HIC-HIC3	0	5.6~5.9%	3.7~4.4%	5.3~5.5%
	11 days	6.3~6.4%	4.5~5.1%	6.2~6.3%
	30 days	6.3~6.4%	5.2~5.4%	6.1~6.1%
Bioactivity	0	103~107%	96~102%	97~102%
	30 days	100~103%	96~107%	91~99%
Binding activity	0	96~106%	92~103%	103~106%
	30 days	95~102%	90~91%	98~104%

Table S5. Forced degradation studies under oxidative stress (post-reconstitution stability) for pre-change, post-change and reference

Tests		Post-change	Pre-change	Reference
NR-CE	0	98.0~98.3%	97.9~98.0%	98.6~98.9%
	1 day	97.4~97.8%	96.4~96.9%	98.0~98.2%
	2 days	97.5~97.9%	95.8~96.7%	97.5~98.0%
R-CE	0	98.3~98.4%	99.0~99.1%	98.5~98.7%
	1 day	97.8~98.0%	98.5~98.7%	98.7~98.8%
	2 days	97.6~97.8%	98.5~98.9%	98.7~98.8%
SEC-monomer	0	98.7~99.5%	98.8~99.3%	99.6~99.7%
	1 day	99.2~99.3%	97.4~98.5%	99.4~99.4%
	2 days	99.2~99.3%	97.4~98.6%	99.4~99.5%
SEC-aggregate	0	0.5~0.9%	0.7~1.2%	0.3~0.4%
	1 day	0.7~0.8%	1.5~2.6%	0.5~0.6%
	2 days	0.7~0.8%	1.4~2.6%	0.5~0.6%

IEX-acidic	0	10.2~10.9%	7.6~9.0%	10.9~11.5%
	1 day	7.7~7.8%	4.3~5.8%	9.6~10.4%
	2 days	6.2~6.3%	3.3~4.5%	9.0~9.6%
IEX-main	0	82.3~83.8%	81.2~82.4%	86.1~87.1%
	1 day	56.6~58.6%	38.1~44.4%	57.2~58.5%
	2 days	44.3~45.4%	27.5~33.3%	46.3~47.9%
IEX-basic	0	5.6~7.1%	9.8~10.6%	2.0~2.4%
	1 day	33.6~35.6%	49.8~57.4%	31.1~33.2%
	2 days	48.3~49.5%	62.2~69.2%	42.8~44.7%
HIC-HIC1	0	70.0~72.3%	70.3~72.2%	60.8~61.7%
	1 day	69.3~70.3%	67.1~68.8%	62.1~62.7%
	2 days	69.5~70.2%	67.1~68.8%	62.3~62.7%
HIC-HIC2	0	17.1~18.0%	18.2~23.6%	27.1~27.9%
	1 day	16.5~17.5%	19.0~20.9%	24.7~25.3%
	2 days	16.7~17.4%	19.1~21.1%	24.6~25.0%
HIC-HIC3	0	5.6~5.9%	3.7~4.4%	5.3~5.5%
	1 day	5.8~6.0%	4.1~4.3%	5.6~5.9%
	2 days	6.2~6.2%	4.3~4.6%	5.7~6.0%
Bioactivity	0	103~107%	96~102%	97~102%
	1 day	100~110%	98~106%	101~105%
	2 days	103~108%	109~111%	103~111%
Binding activity	0	96~106%	92~103%	103~106%
	1 day	98~105%	91~93%	94~97%
	2 days	88~92%	90~96%	91~104%

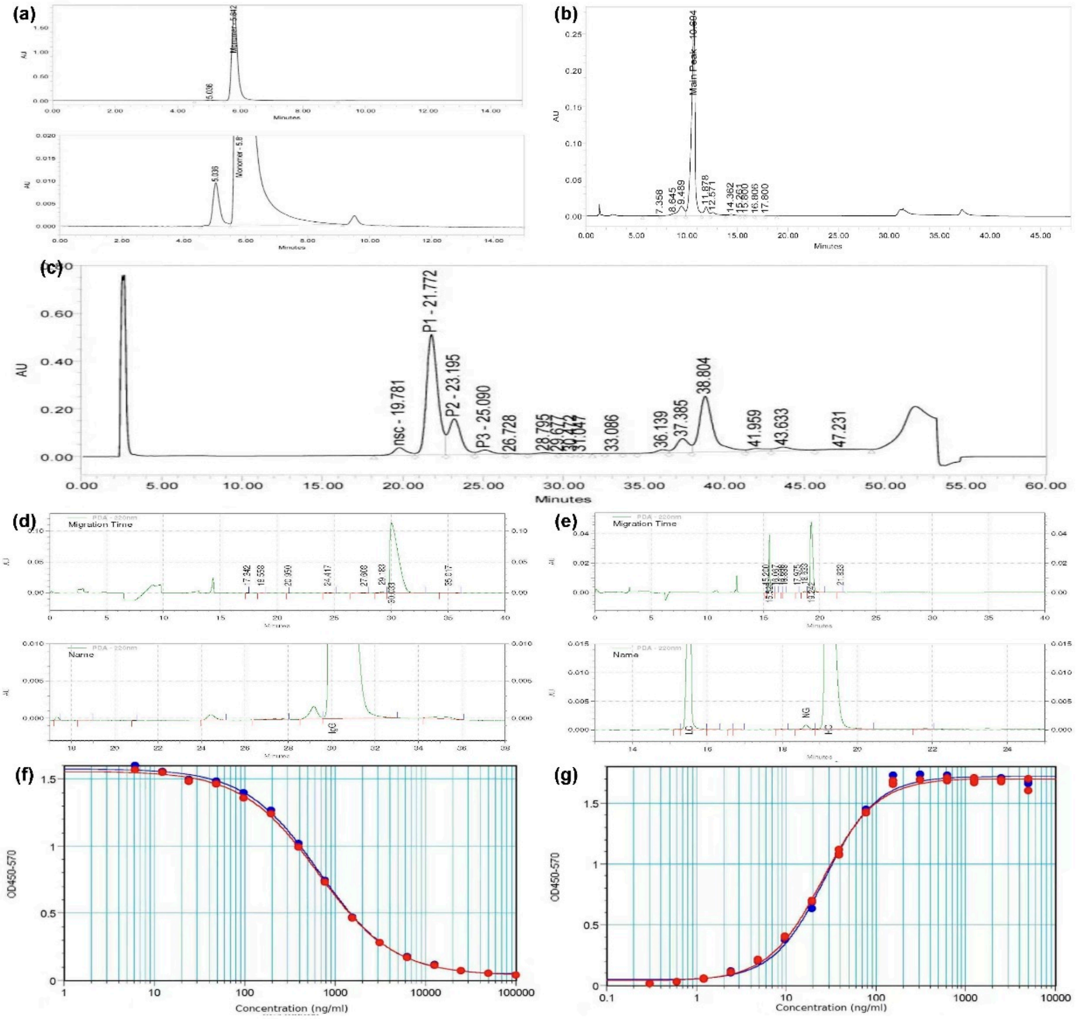

Figure S1. Representative chromatograms/spectra. (a) SEC-UPLC, (b) IEX-UPLC, (c) HIC-UPLC, (d) nrCE-SDS, (e) rCE-SDS, (f) bioactivity, (g) binding activity.

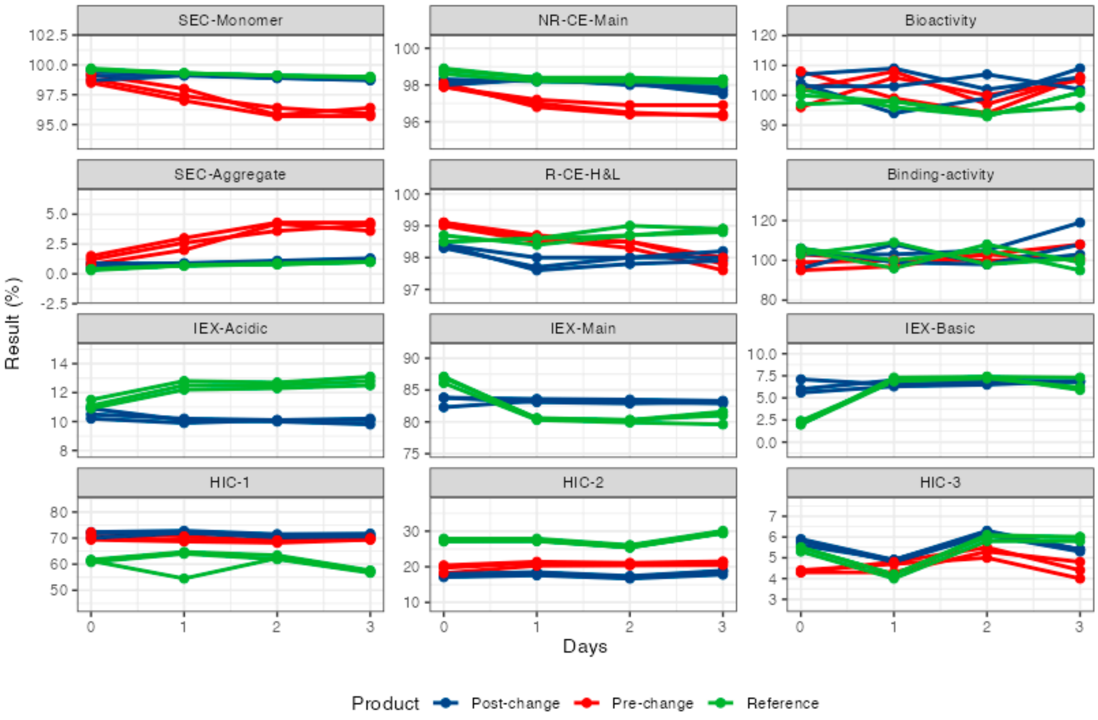

Figure S2. Stability data from accelerated stability studies

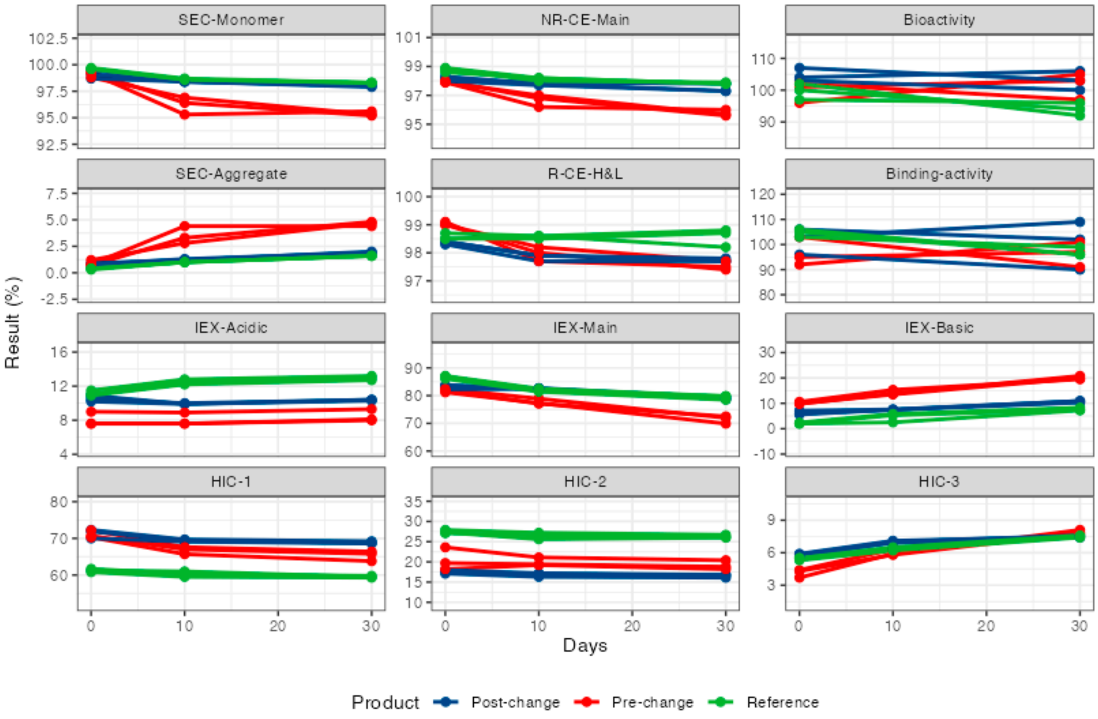

Figure S3. Stability Data from forced degradation under high-temperature stress (40°C)

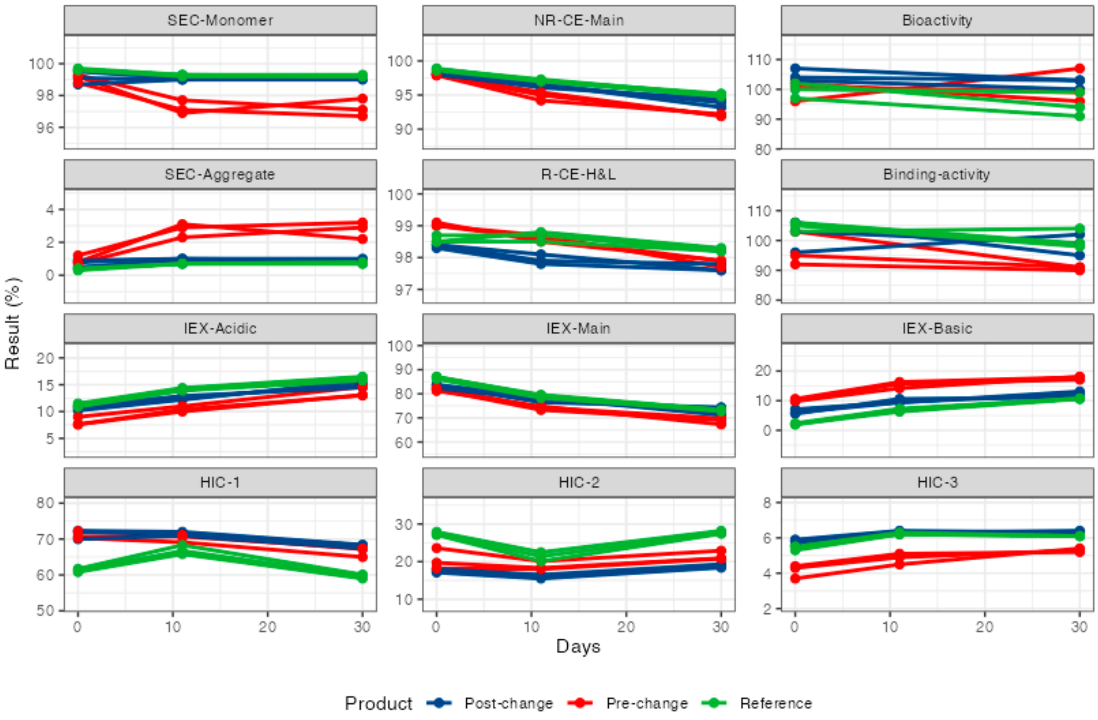

Figure S4. Stability data from photostability testing

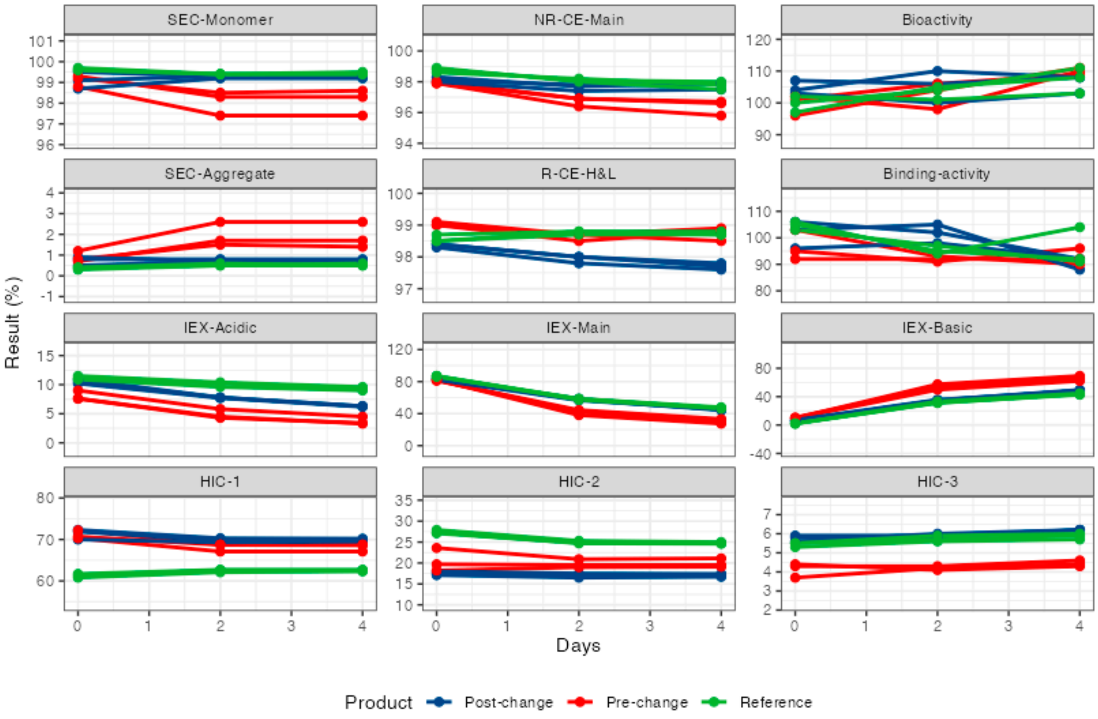

Figure S5. Stability data from oxidative stress testing
